# Hereditary Influences

**Published:** 1872-07

**Authors:** 


### HEREDITARY INFLUENCES.

A SINGLE red ear of corn will sometimes be found in gathering the crop in the autumn, and if one grain of it be planted in the following spring, there will be other red ears. If the grains of all these be planted the next season, it will be a few years only before every grain of corn in the whole field will be red, like the original. By analogy, the same law prevails in living generations, among insects and birds, and animals and man, for "like begets like" throughout the universe of living things. By this general law it follows that if in any community a healthy, intelligent pair should marry, and should in their lives carry out the principles of healthful living laid down in these pages, they will have children; their children will be healthy, intelligent, and prolific, like themselves, each one becoming a centre of population, the progenitor of others, until, in a time not remote, the whole land would be peopled with a stalwart race, possessing physical vigor, active minds, and elevated sentiments; because, in the nature of things, the healthy individuals among animals and men have ability to perpetuate themselves; while the diseased, the weakly, the vicious, die out; for the Scriptures say the wicked "shall not live out half their days;" and just as explicit and positive is the announcement, "The righteous shall go down to his grave like a shock of corn, fully ripe in his season." Hence, the fundamental truth is founded on the eternal rock of Divine assertion, that goodness naturally spreads, perpetuates itself, has in itself the seeds of immortality; while vice and disease have within themselves the seeds of death. Thus far as to the physical man; and not less true is it of the spiritual nature; and on the same rock is the truth grounded, that covenant and mercy would not only be kept towards the good man, but to his children also to thousands of "generations" after him, which means that good men not only transmit their qualities to their own immediate children, but to those born to them to remote ages in the future. And with such responsibilities, with such "exceeding great and precious promises" from the Infinite One, can any good man or woman, can any Christian pair, consistently beget children without any care, without any premeditation, without any arrangement of circumstances to give for good the first impress of physical, mental, and moral character? And yet, the very best men of all ages up to the present hour have habitually committed acts of parentage without the very first thought, without the very slightest deed, intended to have any bearing whatever on the character of the child, but everything has been left to the most perfect hap-hazard imaginable. And no sermon has ever been preached, and no book ever published, nor has any book ever been written, with the express object of enforcing a line of conduct which would give to those who come after us healthy bodies, good constitutions, and mental and moral qualities which would, in the highest sense, fit them for the duties of life. It is a historical fact that "descent," meaning thereby "hereditary influence," does more towards fashioning the physical constitution and the moral character of man than all things else besides, external or internal. And there is scarcely another truth, in the whole range of human observation, which has gained such a universal assent among thinking minds of past ages as that of the hereditary transmission of physical, mental, and moral qualities, and which at the same time has been so universally disregarded in practice; although it should be clear to every one that if a parent becomes addicted to any form of vice, is habitually vicious in any one thing, it cannot fail to leave a bad impress on the child's constitution, and that diseased physical constitutions affect the will and the conscience and the moral nature in such a way as to impair their vigor, and their legitimate, pure, and right action. If any change is to be made in these directions, it must be done by those who have loftier thoughts, higher heroisms, and more transcendent aspirations than have yet influenced mankind. Yet the motives to these higher things may be cherished, may be cultivated in the humblest hearts where true love to God resides, until the morning dawns of a brighter and a better day.

